# Advancing functional foods: a systematic analysis of plant-derived exosome-like nanoparticles and their health-promoting properties

**DOI:** 10.3389/fnut.2025.1544746

**Published:** 2025-03-05

**Authors:** Ke Che, Cong Wang, Hao Chen

**Affiliations:** ^1^College of Food Engineering, Anhui Science and Technology University, Fengyang, China; ^2^Center for Molecular Metabolism, Nanjing University of Science and Technology, Nanjing, China; ^3^College of Life and Health Sciences, Anhui Science and Technology University, Fengyang, China; ^4^Planting Department, Jiuhua Huayuan Pharmaceutical Co., Ltd., Chuzhou, China; ^5^School of Pharmacy, Anhui University of Chinese Medicine, Hefei, China

**Keywords:** plant-derived exosome-like nanoparticles (PDENs), food nutrition and health, bioactive components, stability, functional foods

## Abstract

Plant-derived exosome-like nanoparticles (PDENs), emerging as novel bioactive agents, exhibit significant potential in food science and nutritional health. These nanoparticles, enriched with plant-specific biomolecules such as proteins, lipids, nucleic acids, and secondary metabolites, demonstrate unique cross-species regulatory capabilities, enabling interactions with mammalian cells and gut microbiota. PDENs enhance nutrient bioavailability by protecting sensitive compounds during digestion, modulate metabolic pathways through miRNA-mediated gene regulation, and exhibit anti-inflammatory and antioxidant properties. For instance, grape-derived PDENs reduce plasma triglycerides in high-fat diets, while ginger-derived nanoparticles alleviate colitis by downregulating pro-inflammatory cytokines. Additionally, PDENs serve as natural drug carriers, with applications in delivering therapeutic agents like doxorubicin and paclitaxel. Despite these advancements, challenges remain in standardizing extraction methods (ultracentrifugation, immunoaffinity), ensuring stability during food processing and storage, and evaluating long-term safety. Current research highlights the need for optimizing lyophilization techniques and understanding interactions between PDENs and food matrices. Furthermore, while PDENs show promise in functional food development—such as fortified beverages and probiotic formulations—their clinical translation requires rigorous pharmacokinetic studies and regulatory clarity. This review synthesizes existing knowledge on PDENs’ composition, biological activities, and applications, while identifying gaps in scalability, stability, and safety assessments. Future directions emphasize interdisciplinary collaboration to harness PDENs’ potential in combating metabolic disorders, enhancing food functionality, and advancing personalized nutrition strategies.

## Graphical abstract

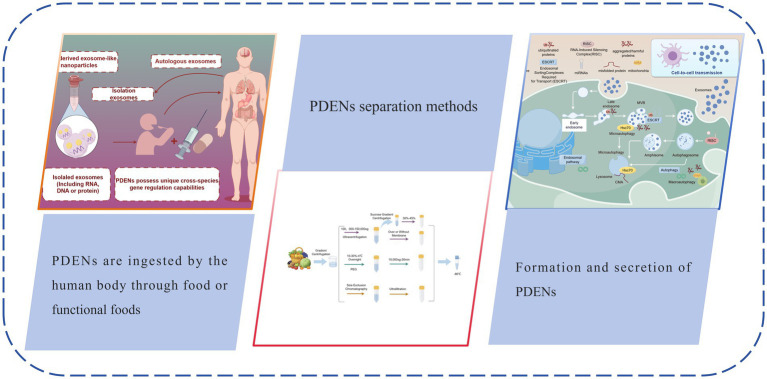

## 1 Introduction

Exosomes, a class of nano-sized extracellular vesicles central to cellular biology, are released into extracellular space from intracellular multivesicular bodies (MVBs) with a refined diameter of 40 ~ 100 nm (1). These intricate vesicles, encapsulated by a lipid bilayer membrane and containing crucial biomolecules such as proteins, RNA, and DNA, play not only a vital role in intercellular communication but also an indispensable part in modulating immune responses and participating in disease processes (2). As our understanding of exosome functions deepens, their potential applications in food science, particularly in the field of nutritional health, are increasingly being explored and valued.

Plant-derived exosome-like nanoparticles (PDENs) unlike exosomes derived from animal or other biological sources, PDENs are characterized by their unique molecular composition, which includes plant-specific bioactive metabolites, proteins, lipids, and nucleic acids (3). These components not only facilitate intercellular communication within plants and enhance their defense mechanisms against pathogens but also enable PDENs to regulate metabolic phenotypes in recipient cells. PDENs mainly originate from vacuoles, endoplasmic reticulum, and Golgi apparatus in plant cells, and are generated through the classical endosome pathway. Their components are rich in plant specific secondary metabolites (such as polyphenols and flavonoids), which play an important role in regulating plant environment interactions, mediating stress responses, and plant microbe interactions (4). They also have significant immune regulation and tissue repair functions in the human body. In contrast, animal derived exosomes are mainly generated through endocytic pathways, consisting mainly of lipids, proteins, and nucleic acids, with functions focused on intercellular signaling, tissue repair, and disease regulation. Scientific interest in exosomes has been growing, especially given their immense potential in mediating intercellular communication across different species. For example, studies have found that exosomes extracted from grapes can be taken up by intestinal cells in mice (5). After entering mouse intestinal cells, these PDENs contain specific miRNAs that can regulate the expression of related genes in mouse intestinal cells, affecting the metabolic activity of intestinal cells. This process demonstrates that plant derived PDENs cross species boundaries and achieve communication with animal cells. In addition, in a study on cross species communication between plants and microorganisms, it was found that PDENs secreted by plant roots can be received by beneficial microorganisms in the rhizosphere soil. The signaling molecules carried by PDENs alter the metabolic pathways of microorganisms, causing them to produce more metabolites that contribute to plant growth, thereby promoting plant growth and health. These specific examples fully demonstrate the enormous potential of PDENs in mediating cell communication between different species.

In the realm of food nutrition and health, the application potential of PDENs is prominently demonstrated in three key dimensions: (1) as carriers of bioactive components, PDENs can protect sensitive nutritional elements from degradation by the digestive system, thereby improving their bioavailability (6), (2) PDENs demonstrate significant biological effects in regulating gut microbiota (7), enhancing immune function (8), and exhibiting anti-inflammatory and antioxidant properties (9), and (3) the nanoscale size and surface characteristics of PDENs make them an ideal drug delivery platform, providing new strategies for developing novel functional foods and nutritional supplements.

Despite the enormous potential of PDENs in food nutrition and health, many unknowns remain regarding their applications in food and mechanisms of action. For instance, issues such as extraction and purification techniques for PDENs, their stability and processing conditions, as well as their bioavailability and safety in the human body require further investigation. Moreover, the functionality and synergistic effects of PDENs in different food matrices, and how they interact with other food components, are important directions for future research.

**Figure 1:**
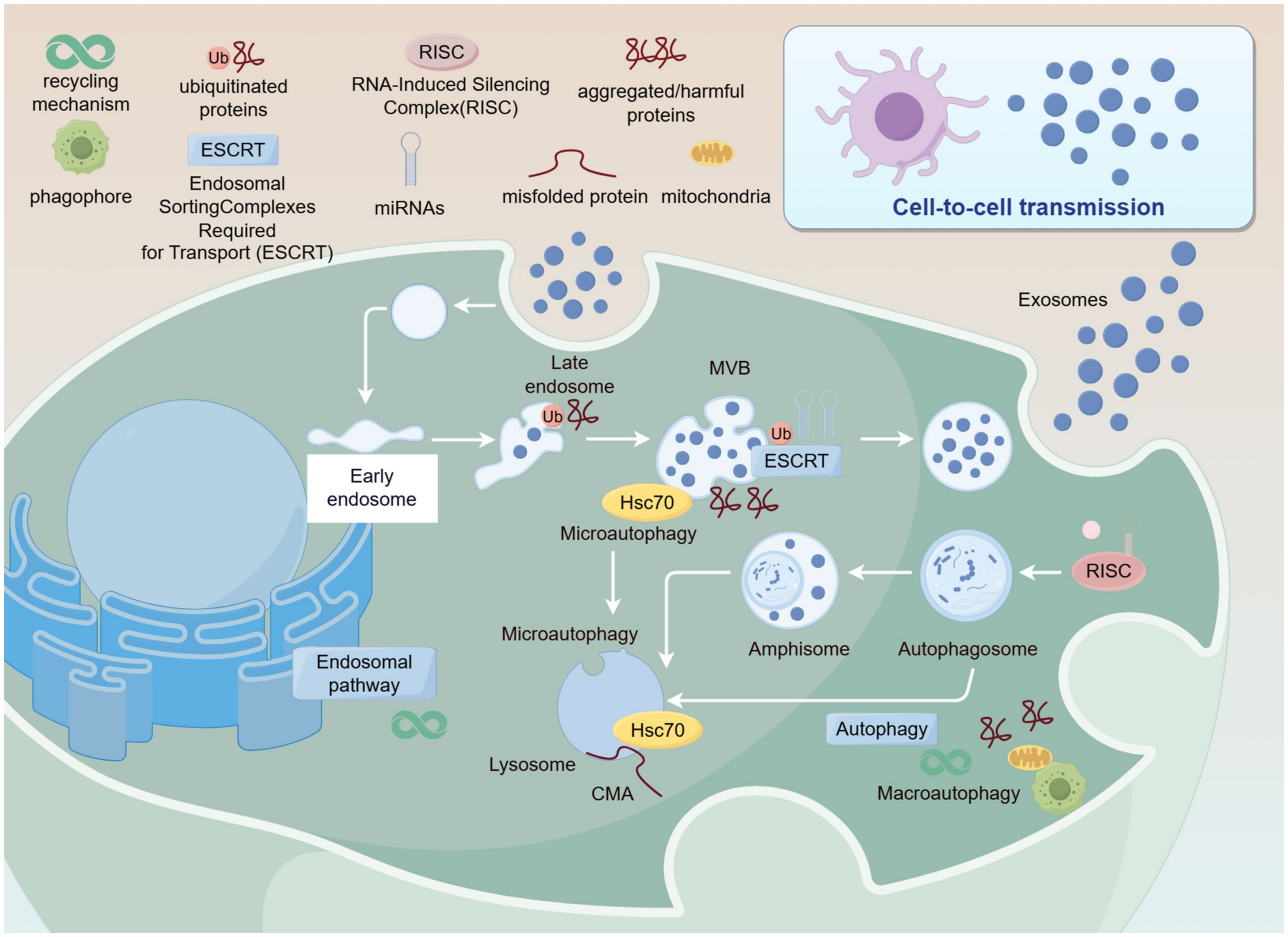
Formation and secretion of PDENs. Created with Figdraw.com.

This review will comprehensively analyze the extraction methods, structural composition, and biological activities of PDENs, as well as their potential applications in food nutrition and health. By examining specific case studies of PDENs in food applications, we will provide readers with a comprehensive perspective to understand how these nanoparticles function as “dark nutrients” in regulating human physiological and pathological processes, and their future development trends in the field of food science (see Figure 1).

## 2 Isolation and characterization of plant-derived exosomes

The isolation and characterization of plant-derived exosomes have become research hotspots in recent years. Plant exosomes play crucial roles in regulating intercellular communication (10) and metabolic processes (11). Multiple isolation and characterization techniques need to be combined to identify plant-derived exosomes, ensuring the acquisition of high-purity, intact exosome samples, thereby laying the foundation for research in molecular regulation and clinical applications. Establishing standard operating procedures is also a direction worthy of attention.

### 2.1 Isolation of plant-derived exosomes

Appropriate extraction methods are crucial for subsequent analysis of exosomes. Methods for isolating Plant-Derived Exosome-like Nanoparticles (PDENs) include ultracentrifugation (12), size-based isolation (13), immunoaffinity (14) and precipitation (15). These four methods are detailed below.

#### 2.1.1 Ultracentrifugation

This method separates exosomes through multiple high-speed centrifugation steps. Ultracentrifugation is the most commonly used (16) technique due to its simplicity, ease of use, long-term affordability, moderate time consumption, and lack of complex sample preparation. First, differential centrifugation is used to remove cells and cellular debris, followed by density gradient centrifugation to further purify the exosomes (17). This method can separate particles based on their density, size, and shape, making it currently the most widely used exosome isolation technique in laboratories.

#### 2.1.2 Size-based isolation

This method utilizes the specific size range of exosomes to separate them using physical barriers (such as filtration membranes) (18). Ultrafiltration is a commonly used technique that employs membranes with different pore sizes to separate various components in the sample (17). Size exclusion chromatography (SEC) uses a porous stationary phase to separate molecules based on their size (19).

#### 2.1.3 Immunoaffinity

This method uses specific antibodies to recognize and capture unique antigen proteins on the exosome surface (20). By immobilizing antibodies on magnetic beads or other carriers, exosomes containing the target antigens can be specifically adsorbed (21). The advantage of this method is its high specificity, but its application may be limited due to the heterogeneity of surface markers on exosomes.

#### 2.1.4 Precipitation

**Figure 2:**
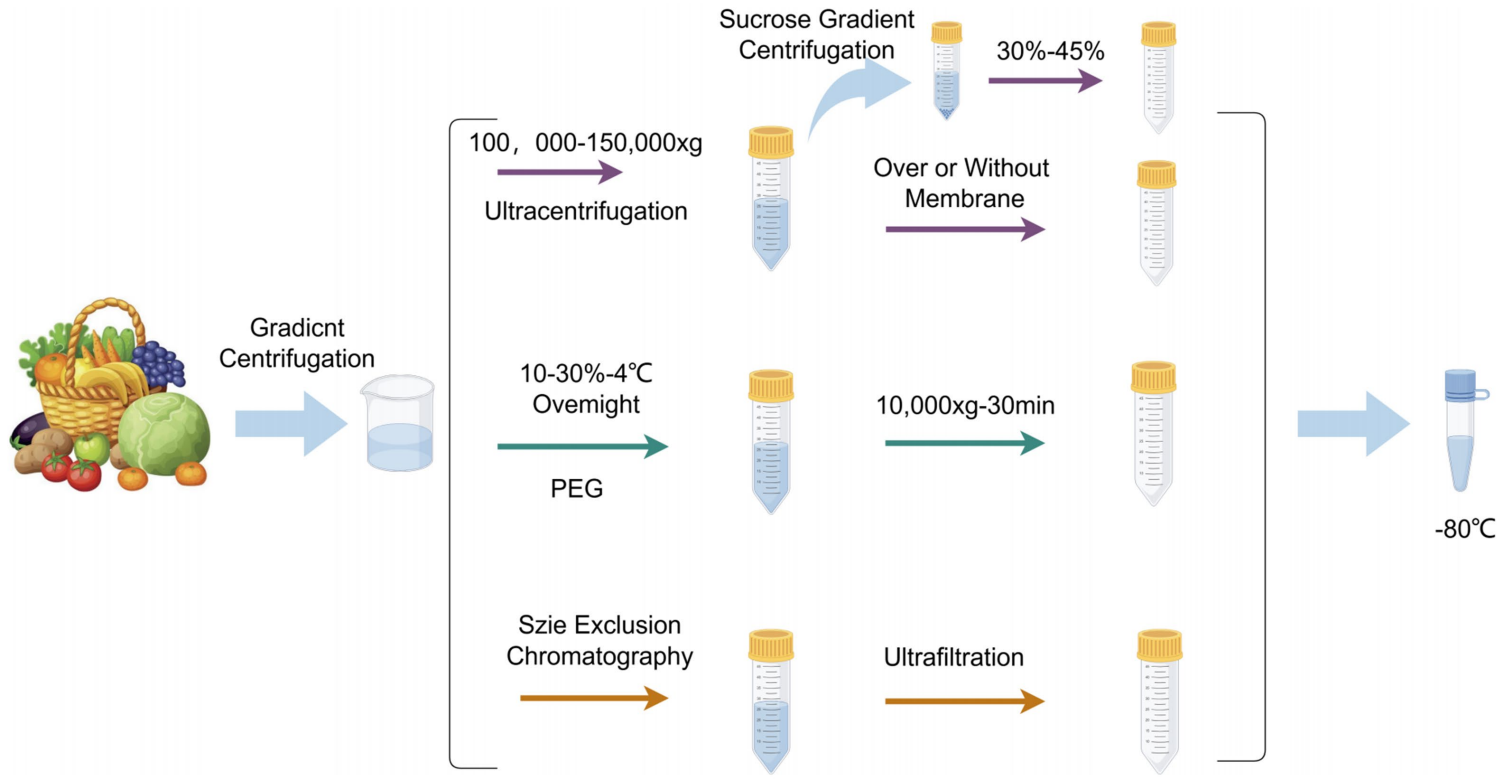
PDENs separation methods. Created with Figdraw.com.

This method uses chemical precipitants (such as polyethylene glycol PEG6000) to promote the aggregation and precipitation of exosomes (22). This method is cost-effective and easy to operate, but may capture non-specific proteins and other particles, thus affecting purity (see Figure 2).

### 2.2 Characterization methods for PDENs

In the process of in-depth research on PDENs, scientists have employed various characterization techniques to reveal their physical and chemical properties. These techniques can be broadly categorized into optical analysis, microscopic imaging, and molecular detection.

#### 2.2.1 Optical analysis techniques

Optical analysis techniques, such as Dynamic Light Scattering (DLS) (23) and Nanoparticle Tracking Analysis (NTA) (24), primarily focus on particle size distribution and Brownian motion, suitable for evaluating the size and concentration of PDENs (25). These methods are quick and convenient but have certain requirements for sample monodispersity. For example, in a study on exosomes isolated from *Arabidopsis thaliana* leaves (26), DLS was used to determine the particle size distribution by measuring the change in scattered light intensity over time (27). This method is suitable for analyzing nanoparticle sizes but requires samples to have a certain level of monodispersity. NTA measures the size and concentration of nanoparticles by directly tracking the Brownian motion of individual particles (28). In the characterization of exosomes from tomato plants (29), NTA provided detailed information about particle size and concentration but has higher requirements for sample purity.

#### 2.2.2 Microscopic imaging techniques

Microscopic imaging techniques, including Transmission Electron Microscopy (TEM) (30), Scanning Electron Microscopy (SEM) (30), and Atomic Force Microscopy (AFM) (31), provide intuitive images of PDENs’ morphology and size. These techniques require special sample treatment, such as staining and drying, but can provide high-resolution surface topography information. In the study of exosomes from rice (32), TEM and SEM provided intuitive images of exosome morphology and size (33). AFM uses the interaction force between a probe and the sample surface to obtain surface topography information. AFM was applied to analyze exosomes from wheat (34), providing high-resolution surface images and analyzing particles under near-physiological conditions (35). However, it should be noted that SEM sample preparation may alter exosome morphology, and the electron beams used in these techniques may damage the exosomes.

#### 2.2.3 Molecular detection techniques

Molecular detection techniques, such as Polymerase Chain Reaction (PCR) (36), Western Blot (37), Mass Spectrometry (38), and Enzyme-Linked Immunosorbent Assay (ELISA) (39), focus on the detection and quantification of specific molecules in PDENs. These methods are crucial for revealing the bioactive components of PDENs. PCR was used to detect and quantify the presence of DNA or RNA molecules in PDENs isolated from maize, detecting specific nucleic acid sequences (40). For example, microRNAs (miRNAs) are typically detected in PDENs using PCR based methods. These miRNAs play an important role in regulating gene expression in receptor cells. Western Blot detects the presence and size of proteins using specific antibodies (41). In the analysis of exosomes from soybean, Western Blot was used to identify and quantify specific protein markers. Heat shock proteins (HSPs), such as HSP70 and HSP90, are common protein markers in PDENs. Specific antibodies against these heat shock proteins were used for Western blot experiments. These heat shock proteins are involved in maintaining the stability of proteins in PDENs and may also play a role in the interaction between PDENs and receptor cells. Mass spectrometry is used to identify and quantify protein and lipid molecules (42). Mass spectrometric analysis of PDENs from tobacco provided detailed information about the protein and lipid composition. Phospholipids, such as phosphatidylcholine (PC), phosphatidylethanolamine (PE), and phosphatidylglycerol (PG), are important lipid components in PDEN. In addition, proteins with unique amino acid sequences can be identified by mass spectrometry. ELISA, a technique based on antigen–antibody reactions used to detect and quantify specific proteins (43). For example, proteins such as lectins can mediate the binding of PDEN to target cells, which can be detected using ELISA with anti lectin antibodies. One of the most important components, which is produced by phospholipase D (PLD) and PLC/DGK using PC, PE, and PG as substrates.

#### 2.2.4 Emerging technologies

In addition, Tunable Resistive Pulse Sensing (tRPS), as an emerging technology, analyzes particle size and charge by measuring the resistance changes caused by particles passing through micropores (44). tRPS is a high-precision technique that can provide detailed information on particle size and charge. In a recent study on exosomes from cucumber, tRPS was successfully applied to analyze the particle size and charge, showing its great potential in plant-related PDENs research (45).

By comprehensively applying these characterization techniques, researchers can gain a thorough understanding of the physical properties, chemical composition, and biological activity of PDENs. However, each detection technique has certain limitations in detecting exosome purity, necessitating further challenges in characterizing exosome purity. As research on PDENs progresses, the demand for achieving clinical treatment purity levels is becoming increasingly stringent.

## 3 Composition of PDENs

PDENs primarily consist of biomolecules including lipids, proteins, nucleic acids, and bioactive compounds (46). Each component of exosomes has its function in terms of biological activity; for instance, the protein spectrum determines the uptake mechanism (47), lipids are essential for effective cellular absorption (48), and miRNAs alter gene expression in cells that absorb PDENs (49). However, no study has yet reported the differences and similarities in miRNA content of PDENs derived from the same fruit or vegetable but of different varieties.

### 3.1 Lipids

PDENs contain various lipid components, which are crucial for maintaining the structure and function of exosomes. The lipid components mainly include phospholipids, triglycerides, and cholesterol, which form the membrane structure of exosomes (50). These lipid components play important roles in the biogenesis, release, targeting, and cellular uptake processes of exosomes (51).

The lipid composition of plant exosomes shows similarities to that of animal-derived exosomes, primarily containing various phospholipids, triglycerides, and cholesterol (52). These lipids not only provide a critical structural foundation for exosomes but also play important roles in various biological functions of exosomes. Notably, another major class of lipids-glycolipids-exists in plant cell membranes (53). The most common among these include digalactosyldiacylglycerol (DGDG), monogalactosyldiacylglycerol (MGDG), and monogalactosylmonoacylglycerol (MGMG) (54). Research has found that ginger contains high levels of DGDG and MGDG (55). DGDG and MGDG may be one of the important factors for the stability of plant exosome-like nanoparticles. Furthermore, studies have found that DGDG derived from oat plant exosome-like nanoparticles can affect the exosome pathway of dectin-1 through the hippocalcin (HPAC)-*β*-glucan pathway (56). It is worth mentioning that dectin-1, as a β-glucan pattern recognition receptor, plays a key role in antifungal immune responses (57). These findings not only deepen our understanding of the lipid components of plant exosomes but also provide important clues for further exploration of their potential applications in immune regulation and disease prevention and treatment.

Lipid components play important roles in intercellular communication, not only helping to maintain cellular homeostasis but also potentially playing important roles in disease prevention and treatment. Plant exosomes have shown great potential in the field of drug delivery due to their higher bioavailability and lower immunogenicity (58). Notably, the lipid components in plant exosomes may be closely related to their various functions such as anti-inflammatory, antioxidant, and anti-tumor effects. These functions not only enrich our understanding of plant exosomes but also open up new possibilities for future medical applications and functional foods. However, although the lipid components of plant exosomes are of significant importance in biology and applications, our understanding of their detailed composition and functional mechanisms remains limited. This field requires more in-depth and comprehensive research to fully reveal the potential of plant exosome lipid components and lay a solid foundation for future functional foods.

### 3.2 Proteins

PDENs are rich in various proteins that may participate in the biosynthesis, transportation, and intercellular communication of exosomes. Protein components include cytoplasmic proteins, transmembrane proteins, and membrane proteins that may serve as channels or transport proteins (59). These protein components are essential parts of plant exosome functions, participating in various biological processes, including intercellular communication and signal transduction.

Proteomic analysis is a widely employed technique that offers dual benefits: it can unveil identifiable markers and elucidate the functions of a specific sample. Consequently, numerous studies have conducted thorough examinations of the proteome within PDENs. The proteome of nanovesicles derived from citrus fruit sacs demonstrated the presence of proteins involved in various processes, including glycolysis (e.g., glyceraldehyde-3-phosphate dehydrogenase), gluconeogenesis (e.g., fructose-bisphosphate aldolase 6), protein folding and transport (e.g., HSP 70, HSP 80, and PTL 39), and cell growth and division (e.g., PTL 3 and clathrin-3). Additionally, enzymes such as hydrolases (e.g., ATPase, pectinesterase, phospholipase, amylase, *β*-galactosidase, and S-adenosylhomocysteine hydrolase) and antioxidants (e.g., SOD, CAT, POD, and GPX) were identified (60).

The presence of these diverse proteins in PDENs suggests their potential involvement in various cellular processes and their ability to influence recipient cells upon uptake. This rich protein composition contributes to the multifaceted functions of PDENs, including their roles in cell–cell communication, metabolic regulation, and potential therapeutic applications. The proteomic analysis of PDENs provides valuable insights into their functional capabilities and potential biological roles. However, further research is needed to fully elucidate the specific functions of individual proteins within PDENs and their interactions with recipient cells. This knowledge will be crucial for understanding the mechanisms by which PDENs exert their biological effects and for developing potential applications in fields such as nutrition, medicine, and biotechnology.

### 3.3 Nucleic acids

MicroRNAs (miRNAs) are part of a class of small RNAs, approximately 22 nucleotides in length, lacking coding characteristics. Their primary function is to regulate gene expression by either facilitating miRNA cleavage or inhibiting miRNA translation, thereby inducing the expression of specific target genes (61, 62). miRNAs can be detected in various bodily fluids through passive leakage and active secretion via membrane vesicles such as exosomes or protein-miRNA complexes (63).

Numerous natural compounds, including curcumin, genistein, EGCG, resveratrol, and quercetin, have been reported to modulate the expression of human miRNAs and confer various health benefits (64–68). For instance, MIR168a from rice, commonly detected in the serum of Chinese subjects, reduces the expression of low-density lipoprotein receptor adapter protein 1 in mouse liver, consequently decreasing the clearance of LDL from mouse plasma (69).

Ginger-derived exosome-like nanoparticles (GELNs) have been shown to be effective in modulating gut microbiota composition. Compared to PBS-treated mice, GELN-treated mice exhibited an increase in Lactobacillaceae and Bacteroidales S24-7, and a decrease in Clostridiaceae. Advanced sequencing techniques applied to GELN RNA have unveiled the presence of at least 109 mature miRNAs within these structures, each possessing the potential to target a wide array of bacterial miRNAs. A noteworthy finding emerged from a mouse model of inflammatory bowel disease, specifically DSS-induced colitis. In this model, researchers identified ath-miR167a, which demonstrated the ability to bind to the mRNA encoding the pilin protein SpaC in *Lactobacillus rhamnosus*. This binding interaction led to the downregulation of SpaC expression, resulting in two significant outcomes: a decrease in the bacterium’s translocation to peripheral blood and a concurrent enhancement of its persistence on the mucosal surface (70).

In addition to miRNAs, PDENs may also contain other types of non-coding RNAs, such as long non-coding RNAs (lncRNAs) and circular RNAs (circRNAs), which play equally important roles in regulating gene expression and cellular functions (71).

Long non-coding RNAs (lncRNAs) are RNA molecules with a length exceeding 200 nucleotides and do not encode proteins (72). They are involved in a wide range of biological processes, including chromatin remodeling, transcriptional regulation, and post-transcriptional regulation. In plant-derived exosomes, lncRNAs can act as molecular scaffolds, bringing together different proteins and nucleic acids to form regulatory complexes. For instance, some lncRNAs in plant exosomes may interact with transcription factors, enhancing or inhibiting the transcription of target genes related to plant-host interactions or stress responses. In a study on plant-pathogen interactions, it was found that certain lncRNAs in plant exosomes can be transferred into pathogens, interfering with their normal gene expression and reducing their virulence.

Circular RNAs (circRNAs) are a unique class of non-coding RNAs formed by the covalent cyclization of exon or intron sequences (73). Due to their circular structure, they exhibit high stability, protecting them from exonuclease-mediated degradation. In plant-derived exosomes, circular RNAs can function as miRNA sponges. They have multiple miRNA-binding sites, enabling them to sequester miRNAs and prevent their binding to target mRNAs. In this way, circular RNAs can indirectly regulate gene expression at the post-transcriptional level. For example, a specific circular RNA was discovered in the exosomes of a certain plant, which can sponge several miRNAs that target genes related to plant growth and development. By sequestering these miRNAs, the circular RNA promotes the expression of these growth-related genes. When the exosomes are taken up by recipient cells, it may affect the overall development of the host organism.

### 3.4 Bioactive compounds

Depending on their plant origin, PDENs may contain specific bioactive compounds such as polyphenols, vitamins, and minerals, which offer various health benefits to the human body.

PDENs contain a wide range of naturally occurring metabolites that may contribute to their therapeutic effects or health-promoting properties in human nutrition: compared to ginger slices, ginger-derived nanovesicles contain highly enriched 6-gingerol, 8-gingerol, 10-gingerol, and 6-shogaol. Thin-layer chromatography detected a significant reduction in shogaols from nanovesicles derived from ginger extract (74).

Broccoli-derived nanovesicles contain sulforaphane. HPLC analysis showed that sulforaphane was more enriched in nanoparticles than in microparticles, while broccoli extract contained almost no free form of sulforaphane (75). Grapefruit-derived nanovesicles contain naringin (76). Nanovesicles derived from citrus lemon and strawberry contain vitamin C (77, 78). Nanovesicles derived from edible tea tree flowers are rich in epigallocatechin gallate, epicatechin gallate, epicatechin, vitexin, myricetin-3-O-rhamnoside, kaempferol-3-O-galactoside, and myricetin (79). Nanovesicles from citrus lemon contain citrate and vitamin C (80). Furthermore, oat-derived nanovesicles contain a five-fold higher percentage of beneficial fiber *β*-glucan compared to oat flour (81), and lemon-derived nanovesicles contain galacturonic acid-rich pectic polysaccharides as active factors (82). This suggests that PDENs could be developed as nano-sized formulations of prebiotics.

In researching and applying PDENs, understanding their composition is crucial for assessing their potential as functional food ingredients or drug delivery systems. As research progresses, we may discover more unknown bioactive components in PDENs, further expanding their application prospects in food science and nutritional health.

## 4 Biological activities and functions of PDENs

PDENs, as emerging bioactive substances, exhibit biological activities and functions similar to animal-derived exosomes, demonstrating diverse functionalities and broad application prospects. They possess unique cross-species gene regulation capabilities, which contribute to their remarkable diversity in health-related functions. PDENs can be utilized not only for developing functional foods with specific health benefits but also as novel food additives and delivery systems for nutritional components. We have compiled preliminary mechanistic studies and health benefits on the biological functional properties of PDENs in recent years (Table 1).

**Table 1:** Biological functional mechanism of action of PDENs.

Plant source	Separation method	Omics	Mechanism of action	Health benefits	references
Ginger	SEC	Proteins, lipids, miRNA	By regulating the Nf2 signaling pathway, significant enhancement of immune function, reduction of oxidative and anti-inflammatory effects can be achieved. Chronic inflammation.	Adjustable proliferation and apoptosis related gene table	(138)
Orange	UC	MiRNA、 lipids	It can regulate the expression of genes related to proliferation and apoptosis.	Promote digestive health and improve the balance of gut microbiota.	(139)
Tea	SEC	Proteins, lipids, miRNA	Display the potential to inhibit cancer cell migration.	Showing promising application prospects in anti-cancer research.	(140)
Broccoli	UC	Proteins	Promote intercellular signal transmission and support cell interactions.	It may play a role in assisting the treatment of diabetes and cardiovascular disease.	(141)
Blueberries	UC	Proteins, miRNA	Enhance intercellular signal transmission, promote targeting and delivery efficiency.	Improve intestinal health and alleviate symptoms of chronic diseases.	(142)
Grape	SEC	Proteins, lipids	By regulating cellular stress response, anti-inflammatory effects can be achieved.	Helps improve heart health and reduce the risk associated with cardiovascular disease.	(91)
Balsam pear	SEC	MiRNA	Promote cellular health by activating antioxidant signaling pathways.	Improve blood sugar control and fight against diabetes and obesity.	(143)
Garlic	ATP	lipids	The antioxidant effect is manifested by regulating the NF-κB signaling pathway.	Improve immune function and protect cardiovascular health.	(144)
Cucumber	SEC	Proteins	Promote intercellular communication and alleviate cellular damage caused by oxidative stress.	Improve hydration, contribute to skin health, and alleviate skin inflammation.	(45)
Ginseng	SEC	Proteins	Plays a role in enhancing immunity by regulating inflammatory responses.	May improve fatigue, enhance physical and mental state.	(145)
Grapefruit	UC	Metabolism	P13K/AKT and MAPK/ERK, inhibit tumor growth	Helps with digestive system health, improves skin health and beauty effects	(29)
Lemon	SEC	Proteins	Phosphorylation of ACACA-ERK1/2 and P38-MAPK-anti-tumor effect	Enhance the body’s immune function and fight against tumors	(146)

### 4.1 Anti-inflammatory effects

PDENs have been shown to reduce inflammatory responses. For instance, GLEN can maintain intestinal homeostasis by increasing the expression of anti-inflammatory cytokines (such as HO-1 and IL-10) while reducing the expression of pro-inflammatory cytokines (such as IL-6 and TNFα) (83).

Regarding the development of functional foods that help alleviate chronic inflammation, plant-derived exosome-like nanovesicles have potential value in preventing and improving inflammation-related diseases. Since PDENs primarily localize in the gastrointestinal tract after oral administration and can exert biological effects on this region, their therapeutic effects on colitis have been studied. When orally administered, broccoli-derived nanovesicles carrying sulforaphane activate adenosine monophosphate-activated protein kinase in dendritic cells and prevent dextran sulfate sodium (DSS)-induced colitis (84). Another study showed that in a DSS-induced colitis model, oral administration of ginger-derived nanoparticles downregulated pro-inflammatory cytokines (TNF-*α*, IL-6, and IL-1β) and upregulated anti-inflammatory cytokines (IL-10 and IL-22) (85).

Furthermore, 6-shogaol found in GELNs was discovered to induce Nrf2 nuclear translocation by modulating the TLR4/TRIF pathway, leading to dissociation from Keap1 and subsequent translocation to the cell nucleus. This results in the formation of heterodimers with Maf and further activation of ARE-mediated downstream gene expression, ultimately promoting the repair process in alcoholic liver injury (86). Research has shown that GELN compounds possess the capability to specifically target the NLRP3 inflammasome, leading to notable improvements in inflammatory conditions (87). Comparable mechanisms have been observed in shiitake mushrooms, highlighting the potential widespread nature of this anti-inflammatory action (88). Furthermore, investigators employed a combination of high-throughput sequencing and bioinformatics techniques to predict the target genes of three distinct miRNAs found in blueberry-derived nanovesicles (89). This analysis revealed the potential anti-inflammatory effects of these miRNAs, which appear to operate by modulating reactive oxygen species (ROS) levels through the TNF-*α* signaling pathway.

Overall, PDENs demonstrate diverse mechanisms of action and broad application prospects in the field of anti-inflammation. From regulating cytokine expression to influencing specific inflammatory signaling pathways, PDENs provide rich research directions for developing novel anti-inflammatory strategies. Future research needs to further elucidate the mechanisms of action of PDENs, optimize their preparation methods, and explore their clinical application potential in various inflammatory diseases, laying a solid foundation for the development of anti-inflammatory functional foods.

### 4.2 Supporting the growth of beneficial Gut microbiota

PDENs demonstrate significant effects in modulating the composition of gut microbiota. Studies have shown that PDENs from various sources have multifaceted impacts on intestinal flora and host health: garlic-derived exosomes promote the growth of beneficial bacteria by interacting with *Lactobacillus rhamnosus* in the gut. This effect extends beyond microbial regulation, as it also stimulates the expression of interleukin-22 (IL-22) by activating the aryl hydrocarbon receptor (AHR) signaling pathway, thereby promoting intestinal tissue repair and healing (90). This selective promotion may play a crucial role in maintaining the balance of gut microbiota. PDENs derived from grapes and turmeric have been found to enhance the barrier function of intestinal epithelial cells, thus affecting nutrient absorption and metabolism (91, 92). This effect is primarily achieved by upregulating the expression of tight junction proteins (such as claudin-1 and occludin), thereby strengthening intercellular connections and improving intestinal barrier integrity. PDENs from apples and broccoli may influence the function of intestinal endocrine cells, regulating the secretion of gut hormones such as glucagon-like peptide-1 (GLP-1) (75). GLP-1, as an important intestinal hormone, plays a key role in regulating appetite, promoting insulin secretion, and maintaining overall metabolic balance (93).

However, the mechanisms by which PDENs from different plant sources affect gut microbiota and host health are complex and still under exploration. In general, PDENs can directly interact with bacteria in the gut. They may modify the cell membrane permeability of pathogenic bacteria, making them more vulnerable to the body’s immune defenses. PDENs are known to regulate the functions of pathogenic or intestinal bacteria. For example, nanovesicles derived from lemons have demonstrated the ability to inhibit pathogens, specifically suppressing Clostridioides difficile, a bacterium responsible for causing diarrhea and pseudomembranous colitis. This inhibition is achieved by enhancing the survival of two probiotic strains: *Streptococcus thermophilus* ST-21 and *Lactobacillus rhamnosus* GG (LGG) are two probiotics that work in synergy with PDENs. At the molecular level, PDENs can enhance the adhesion ability of these probiotics to the intestinal epithelial cells. They carry bioactive molecules like small RNAs and proteins which can be transferred to the probiotics. Once inside, these molecules can upregulate the expression of genes related to the synthesis of aryl hydrocarbon receptor (AhR) ligands in the probiotics. These probiotics work in synergy, resulting in increased production of aryl hydrocarbon receptor (AhR) ligands. The key AhR ligands produced include indole-3-lactic acid, indole-3-aldehyde (I3A), and lactic acid, which contribute to the overall beneficial effects observed (94). For instance, the upregulated gene expression in the probiotics due to the influence of PDENs promotes the metabolic pathways that lead to the synthesis of these AhR ligands. Additionally, the interaction between PDENs and probiotics can also modulate the intracellular signaling pathways in the probiotics, further enhancing the production and release of these beneficial ligands. The AhR signaling pathway enhances intestinal IL-22 production and maintains mucosal antimicrobial agent levels, thereby maintaining intestinal barrier function. Using AhR gene knockout mice, IL-22 gene knockout mice, and germ-free mice, it was conclusively demonstrated that ginger-derived nanovesicles are absorbed by the probiotic LGG and induce I3A, which promotes AhR pathway activation and IL-22 production (95). Furthermore, Polyphenols from tea promote the growth of beneficial intestinal bacteria while inhibiting harmful bacteria, thus improving gut health (96). These research findings not only deepen our understanding of the role of PDENs in intestinal health but also provide a basis for developing novel functional foods based on plant exosomes to regulate gut microbiota.

### 4.3 Metabolic regulation

The regulatory effects of PDENs on host metabolism represent an emerging and exciting field of research. These nano-scale structures not only facilitate cross-species communication between plants and animals but also significantly influence various metabolic processes in the host. Below is a detailed exposition of the role of PDENs in metabolic regulation.

#### 4.3.1 Lipid metabolism regulation

Multiple studies have demonstrated that PDENs can effectively reduce blood triglyceride and cholesterol levels. For instance, exosomes derived from grapes have been shown to significantly lower plasma triglyceride content in mice fed a high-fat diet (97). The mechanism may be related to the presence of polyphenolic compounds such as resveratrol in grape exosomes. Resveratrol can activate the AMP-activated protein kinase (AMPK) pathway (98). Activation of AMPK leads to the phosphorylation and inactivation of acetyl-CoA carboxylase (ACC), a key enzyme in fatty acid synthesis. This results in decreased fatty acid synthesis and an increase in fatty acid oxidation, thereby reducing triglyceride levels. Exosomes from fruits and vegetables, in particular, have shown potential in ameliorating non-alcoholic fatty liver disease. These exosomes may achieve this effect by modulating the expression of genes related to hepatic lipid synthesis and oxidation (99). Some PDENs can influence the differentiation and function of adipocytes. For example, curcumin-enriched exosomes can inhibit adipocyte differentiation, potentially impacting weight control (100). Curcumin in these exosomes can downregulate the expression of key transcription factors such as peroxisome proliferator-activated receptor-*γ* (PPAR-γ) and CCAAT/enhancer-binding protein-*α* (C/EBP-α), which are crucial for adipocyte differentiation (101). Ginger exhibits comprehensive and positive effects on metabolic regulation. It can effectively lower blood glucose levels and improve insulin sensitivity. Ginger can also significantly reduce total cholesterol, low-density lipoprotein, and triglyceride levels in the blood, contributing to the prevention of fatty liver disease (102). Animal experiments on rats with hyperlipidemia have shown that ginger extract can reduce lipid levels in the blood. The active components in ginger, such as gingerols and shogaols, may play a role in modulating lipid metabolism by inhibiting lipid synthesis enzymes and promoting lipid-clearing processes (103). Research has found that catechins in tea can significantly reduce blood levels of low-density lipoprotein cholesterol and total cholesterol (104). Green tea extract can also markedly enhance metabolic rate and increase energy expenditure during weight maintenance periods (105). Catechins, especially epigallocatechin-3-gallate (EGCG), can inhibit 3-hydroxy-3-methylglutaryl-coenzyme A (HMG-CoA) reductase, a key enzyme in cholesterol synthesis (106). In animal experiments, mice fed a high-fat diet supplemented with green tea extract showed lower cholesterol levels compared to the control group.

#### 4.3.2 Glucose metabolism regulation

Various plant-derived exosomes can enhance insulin sensitivity. This effect may be achieved by regulating the expression of proteins related to the insulin signaling pathway. Grapes are rich in polyphenolic compounds, such as resveratrol and quercetin, which possess significant antioxidant capabilities (107). Studies have shown that grape extract can improve insulin sensitivity and lower blood glucose levels (108). Resveratrol in grape extract can activate the insulin-like growth factor-1 (IGF-1) receptor, leading to the activation of downstream signaling molecules such as phosphatidylinositol 3-kinase (PI3K) and protein kinase B (Akt). This activation promotes glucose uptake into cells, thus reducing blood glucose levels. In a study on diabetic rats, grape extract supplementation improved glucose tolerance and insulin sensitivity (109). Exosomes from yams can significantly reduce fasting blood glucose levels in diabetic model mice. Research has found that some PDENs can promote glucose uptake in muscle and adipose tissues. This action may be achieved by upregulating the expression or activity of the glucose transporter GLUT4. Yam exosomes may contain bioactive substances that activate the PI3K-Akt-GLUT4 signaling pathway. In a mouse model of type 2 diabetes, treatment with yam exosomes increased the expression of GLUT4 in muscle and adipose tissues, leading to enhanced glucose uptake and reduced blood glucose levels (110).

#### 4.3.3 Energy metabolism regulation

Exosomes from certain tropical fruits, in particular, have been found to increase host energy expenditure. This may be achieved by activating brown adipose tissue or promoting the “browning” of white adipose tissue. Certain miRNAs and small molecular compounds contained in PDENs can regulate the activity of key metabolic enzymes. For example, exosomes from tea leaves can influence the activity of fatty acid synthase and acetyl-CoA carboxylase. Animal experiments on mice have shown that administration of tea exosomes can increase the expression of uncoupling protein 1 (UCP1) in white adipose tissue, promoting the conversion of white adipose tissue into brown-like adipose tissue and increasing energy expenditure (111). Studies have shown that natural compounds of yam polysaccharides and yam proteins exhibit therapeutic effects on metabolic syndrome. This combination can effectively treat metabolic syndrome characterized by obesity, insulin resistance, hypertension, hyperlipidemia, and fatty liver (112). The active components of yams not only improve metabolic indicators but also provide protective effects for the heart and kidneys (113, 114). In a rat model of metabolic syndrome, treatment with yam polysaccharides and proteins reduced body weight, improved insulin sensitivity, and reduced lipid levels in the blood. The specific components in yam polysaccharides and proteins may act on multiple metabolic pathways, such as regulating the expression of genes related to lipid metabolism and insulin signaling (115).

In summary, these plant-derived substances influence host metabolism through various mechanisms, including regulating blood glucose levels and improving lipid metabolism. These research findings provide a scientific basis for developing functional foods and health strategies based on PDENs’ metabolic regulation aspects, showing promise in playing an important role in the prevention and auxiliary treatment of metabolism-related diseases.

### 4.4 As drug delivery systems

The nano-scale dimensions and surface characteristics of PDENs make them potential drug delivery systems (14). For instance, grape-derived exosomes can traverse the intestinal mucus barrier in mice via oral administration, stimulating the proliferation of intestinal stem cells, thereby contributing to the restoration of intestinal structure (116).

Due to their lipid structure being highly similar to that of the plasma membrane, PDENs can protect their contents from external factors. Consequently, they serve as natural nanocarriers for bioactive compounds (117). A significant added value is the high level of resistance of the nanovesicle lipid membrane, which has been demonstrated using ultrasonic treatment. Some examples include ginger-derived nanovesicles, which have been used to deliver doxorubicin using electrostatic interactions (118); and grapefruit nanovesicles loaded with paclitaxel have been successfully administered intranasally in mouse models (119). Both pieces of evidence provide proof of concept, supporting the use of plant-derived nanovesicles as a novel, highly effective, and side-effect-free approach for nanodelivery of therapeutic molecules. There is widespread interest in using plant-derived nanovesicles for drug delivery, which have been shown not to undergo filtration organ sequestration and exhibit limited or no systemic toxicity (29).

The realization of these functions and benefits relies on the integrity of PDENs, particularly the integrity of their lipid bilayer membrane, which is crucial for their health-promoting functions. However, PDENs are sensitive to storage and processing conditions, and their extraction costs are relatively high, factors that limit their potential as functional food ingredients. Future research needs to address the stability issues of PDENs in functional foods and further explore their health benefits and potential clinical applications. This includes gaining a deeper understanding of their mechanisms of action, optimizing extraction and purification methods, and evaluating their stability and efficacy in practical food systems.

## 5 Stability

The stability of PDENs is a crucial factor for their application as functional food ingredients, significantly affecting the accuracy of subsequent analyses and product development and utilization. The stability of PDENs is particularly important during digestion, food processing, and storage, as these factors can influence the integrity of their membranes and biological activity. PDENs demonstrate good stability during the digestive process and can survive and enter the colonic region. This indicates that PDENs have potential bioavailability as functional food ingredients in the human body.

### 5.1 Stability in the digestive environment

Studies have shown that PDENs can maintain their structure in the digestive environment. PDENs only exhibit changes in size and charge during *in vitro* digestion, but their overall structure remains stable (107). When administered orally, PDENs can pass through the digestive process and enter the colonic region (120), indicating that their stability in the digestive environment is sufficient to support their potential as functional food ingredients.

In terms of stability, grapefruit-derived nanovesicles have shown resistance to *in vitro* digestion by gastric pepsin and pancreatic and bile extract solutions (55). The stability of ginger-derived nanovesicles was also tested by incubating them in a gastric-like solution (pepsin solution at pH 2.0) or first in a gastric-like solution and then in a mouse gastric pepsin-like solution (bile extract and pancreatic enzyme solution adjusted to pH 6.5). Results showed that ginger-derived nanovesicles were stable in these solutions, with a slight decrease in size and *ζ*-potential changes according to the surrounding pH: negatively charged in PBS and curcumin-like solutions; slightly positively charged in gastric-like solutions. Turmeric-derived nanovesicles also maintained nanoscale sizes in solutions of different pH, with increased size and experiencing ζ-potential changes similar to ginger-derived nanovesicles (121). These collectively suggest that PDENs, due to the versatility of their membranes, will survive in the harsh environment of the gastrointestinal tract when administered orally in food form or purified form.

### 5.2 Stability in food processing

PDENs need to maintain their membrane integrity during food processing to retain their biological activity. Although current research on the stability of PDENs during food processing is limited, studies on exosomes from other sources provide some reference. For example, exosomes in milk have shown high stability under adverse conditions such as acidification, boiling, and freeze–thaw cycles (122). However, another study showed that miRNA in milk decreased by 63% during pasteurization and homogenization, possibly due to damage to the exosome membrane during processing (123). These findings suggest that exosomes from different sources may exhibit significant differences in stability under processing conditions.

PDENs generally show good stability under freezing conditions. However, repeated freeze–thaw cycles may affect their integrity. For instance, multiple freeze–thaw cycles at −20°Cof human saliva-derived exosomes lead to decreased membrane integrity (124). Mechanical treatments such as homogenization and stirring may also impact the structural integrity of PDENs. However, specific studies on the effects of mechanical processing on PDENs stability are currently limited.

### 5.3 Stability under storage conditions

The stability of PDENs is crucial for their function and application, and storage conditions are key factors affecting PDENs stability. Temperature is one of the most critical factors influencing PDENs stability. Studies have shown that most PDENs exhibit good stability under refrigeration at 4°C. For instance, vesicles derived from ginger and rehmannia can maintain stability for over two weeks at 4°Cwithout significant changes (125). This suggests that 4°C may be an ideal temperature for short-term storage of PDENs. However, the impact of freezing at −80°C for long-term storage remains controversial. Some studies have found that PDENs can remain stable at −80°C, while others indicate that the freezing process may damage the structure and function of PDENs. This discrepancy may be related to the different lipid compositions and membrane structures of PDENs from various sources.

Repeated freeze–thaw cycles have a significant impact on the stability of PDENs. This process involves the disruption and rearrangement of lipid membranes, which may substantially alter the structure and function of PDENs. Therefore, repeated freezing and thawing of PDENs should be avoided in practical applications.

Lyophilization technology is considered a potential method to improve the long-term storage stability of PDENs. If lyophilized PDENs can be stably preserved at room temperature, it would greatly expand their range of applications. Currently, there are some preliminary comparative studies on PDENs in relation to the impact of lyophilization. For example, research on exosomes derived from fruits such as grapes and apples has shown that the chemical composition and structure of exosomes from these two plant sources vary (91, 126). Grapes-derived exosomes, which are rich in polyphenols like resveratrol, seem to be more sensitive to the lyophilization process in terms of maintaining their integrity. During lyophilization, the loss of some bioactive components in grape-exosomes is relatively higher compared to apple-exosomes. This may be due to the unique structure of grape-exosomes and the interaction between resveratrol and other components. On the other hand, apple-exosomes, with a relatively higher content of pectin-like substances, show better stability during lyophilization, maintaining a relatively higher proportion of their original particle size distribution and bioactivity.

However, current research on the stability of lyophilized PDENs is still relatively limited, and more experimental data are needed to comprehensively verify its effectiveness for exosomes from various plant sources, especially in terms of how different plant-derived exosomes respond to lyophilization in relation to their long-term storage stability and subsequent functionality.

The stability of PDENs also varies in different pH environments. For example, the particle size of grapefruit-derived PDENs is more uniform in acidic solutions compared to alkaline solutions (127). Ginger-derived vesicles undergo changes in particle size and surface charge in simulated gastric fluid (pH 2.0) and simulated intestinal fluid (pH 6.5) (128). These findings have important implications for understanding the transport and distribution of PDENs *in vivo*.

Although there are few specialized studies on the effects of humidity on PDENs stability, research has shown that miRNAs in exosomes can remain stable for 48 h under appropriate temperature and humidity conditions. This suggests that humidity may be another important factor affecting PDENs stability, warranting further in-depth research.

**Figure 3:**
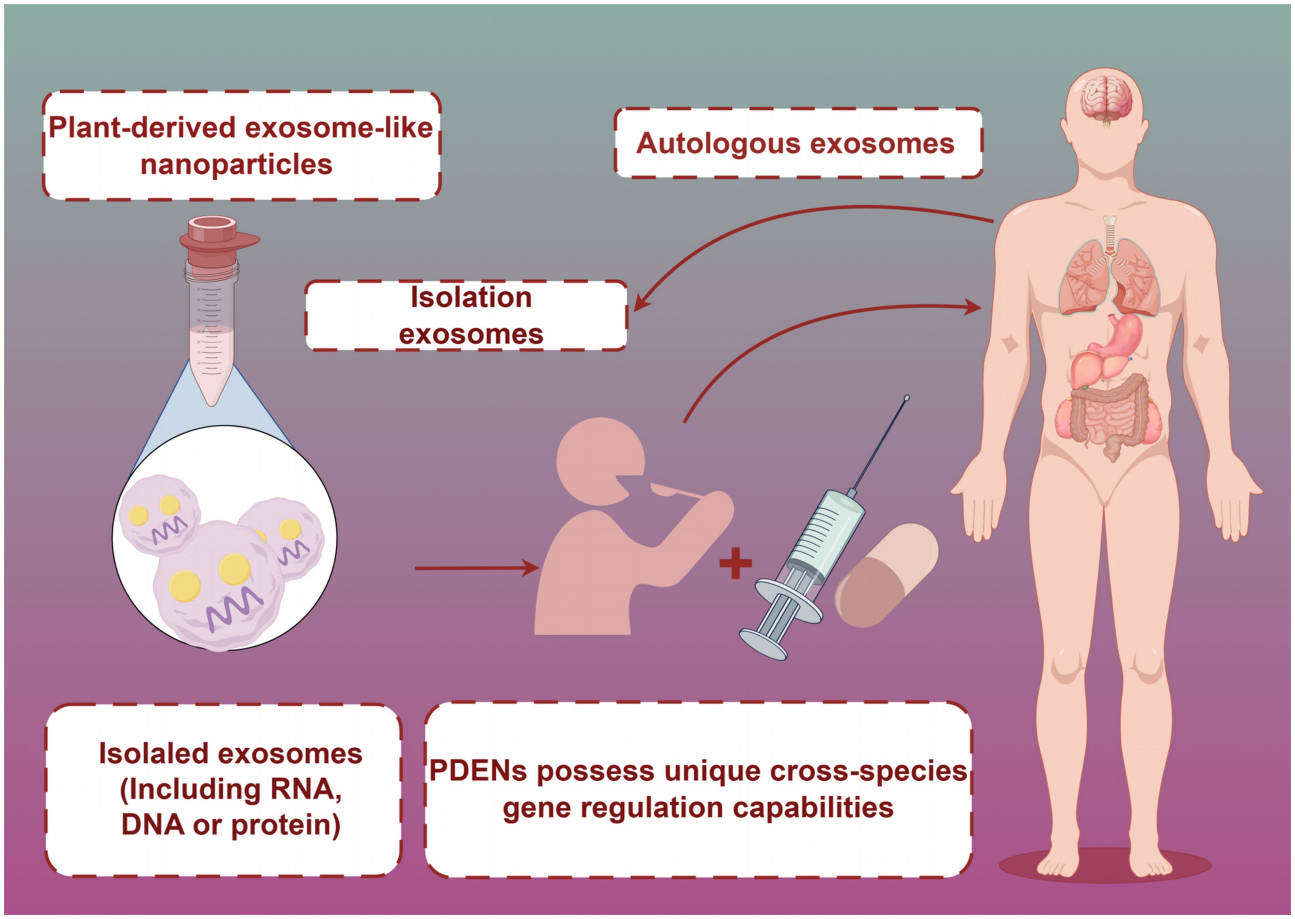
PDENs are ingested by the human body through food or functional foods. Created with Figdraw.com.

Direct studies on the effects of light exposure on PDENs stability are currently lacking. However, considering the lipid membrane structure of PDENs, light exposure may induce photo-oxidation reactions, thereby affecting their stability. This aspect requires more experimental data for support (see Figure 3).

## 6 Future research directions

### 6.1 Research on bioactivity and functionality of PDENs

Future research on PDENs should delve deeper into their bioactivity and functionality to fully realize their application value in food nutrition and health.

Exosomes are rich in various bioactive substances, including proteins, lipids, nucleic acids, and secondary metabolites, endowing them with multiple therapeutic capabilities (53). Future research should concentrate on comprehensively identifying and characterizing these active components. For example, grape-derived exosomes might contain unique polyphenolic compounds with potent antioxidant properties, while ginger-derived exosomes could have specific bioactive components with anti-inflammatory effects. In-depth studies on the biosynthetic pathways and regulatory mechanisms of these active components are needed to enable targeted modification and enrichment of specific active substances.

Research on the functionality of PDENs should be a priority. PDENs have been shown to possess functions such as hepatoprotection, anti-tumor, antioxidant, anti-inflammatory, and tissue regeneration effects (129–132). Further exploration of the underlying mechanisms is essential, especially at the molecular and cellular levels. For example, grape-derived exosomes may induce intestinal stem cell proliferation and alleviate colitis through the activation of specific signaling pathways, which requires in-depth research (132). The scope of functional research on PDENs should also be expanded to explore their potential roles in immune regulation, metabolic control, and neuroprotection.

Investigating the *in-vivo* bioavailability of PDENs is a crucial future direction. Although plant exosomes can penetrate the intestinal mucus layer and target intestinal stem cells, their absorption, distribution, metabolism, and excretion processes in the human body remain to be thoroughly studied (133). Systematic pharmacokinetic and biodistribution studies should be carried out to clarify the fate of plant-derived exosomes in the body, providing a scientific basis for their application in functional foods.

Exosomes, with their small size, strong tissue penetration capabilities, and good physicochemical stability under different pH and temperature conditions, are ideal drug delivery carriers. The focus should be on enhancing their targeting ability, such as modifying their surface with specific ligands or using genetic engineering to express targeting peptides, to increase the precision and efficiency of drug delivery.

### 6.2 Stability studies under storage conditions

Research on the stability of PDENs under diverse storage conditions remains inadequately explored and demands further in-depth investigation. Future studies should zero in on evaluating how storage factors impact the structural integrity, biological activity, and functionality of plant-derived exosomes.

A systematic examination of the mechanisms by which environmental factors like temperature, humidity, and light exposure influence exosome stability is essential. For instance, extreme temperature fluctuations could potentially disrupt the lipid bilayer of exosomes, leading to the leakage of their bioactive contents. Different humidity levels might also affect the hydration state of exosomes, altering their physical properties.

Exploring the impact of preservation methods such as lyophilization and spray drying on exosome physicochemical properties is another crucial aspect. Lyophilization, for example, might cause shrinkage or aggregation of exosomes if not properly optimized, while spray drying could potentially lead to the degradation of heat-sensitive bioactive components. Additionally, the influence of packaging materials, like plastic polymers or glass containers, and packaging methods, such as vacuum-sealed or air-permeable packaging, on exosome stability needs to be investigated.

Long-term storage experiments are vital for assessing the shelf life of plant-derived exosomes, which will offer a scientific basis for their application in the food industry. Moreover, establishing rapid and accurate quality assessment methods, such as nanoparticle tracking analysis (NTA) for size distribution and enzyme-linked immunosorbent assay (ELISA) for bioactive component quantification, is significant for monitoring stability changes during storage.

Future research should aim to: establish standardized storage conditions and evaluation systems for PDENs, taking into account factors like optimal temperature, humidity, and packaging materials. Conduct in-depth investigations into the stability differences among PDENs from various sources under different storage conditions and their underlying mechanisms. For example, exosomes from tropical fruits might have different stability profiles compared to those from temperate-climate plants. Explore novel storage technologies, such as the use of cryoprotectants in combination with lyophilization techniques for long-term storage of PDENs. Study the effects of storage conditions on the biological functions of PDENs, not just their physicochemical properties. This could involve assessing the ability of stored exosomes to still modulate cellular signaling pathways.

As research advances, we will gain better understanding and control over the stability of PDENs. These studies will not only enhance the application potential of plant-derived exosomes in food health and nutrition but also provide a theoretical foundation and technical support for the development of novel functional foods.

### 6.3 Stability studies during food processing

Investigating the stability of PDENs under different food processing conditions, especially the impact of thermal and mechanical treatments on their membrane integrity and biological activity, is of utmost importance.

pH is a significant factor influencing the stability of plant-derived exosomes. For example, in acidic pH conditions, the surface charge of exosomes might change, leading to aggregation or dissociation of their membrane components. In a study on the development of exosome-containing functional beverages, it was found that exosomes were more stable within a pH range of 6–8 (134).

Temperature is another critical factor. High-temperature processing, such as pasteurization or sterilization, can cause the denaturation of exosomal proteins and the rupture of the lipid membrane, resulting in the release of contents. Precise control of processing temperature and duration is necessary to retain maximum biological activity. For instance, a short-time, high-temperature (STHT) treatment might be more effective in preserving exosome integrity compared to long-term, low-temperature processing.

Light exposure and oxidation also play crucial roles. Prolonged light exposure can lead to the degradation of certain bioactive substances in exosomes, such as miRNA, which is essential for their regulatory functions (135). The presence of oxygen can accelerate the oxidation of exosome membrane structures, reducing their stability. Therefore, during food processing, storage, and transportation, measures like vacuum packaging or the addition of appropriate antioxidants, such as tocopherols, should be considered. Additionally, certain food additives like ascorbic acid (VC) may help improve exosome stability, but further research and validation are required (136).

Processing techniques also have a notable impact on plant-derived exosome stability. High-pressure processing technology can maintain exosome structural integrity better than traditional thermal treatment in some cases (137). Microencapsulation technology is a promising method to enhance exosome stability. By encapsulating exosomes in a protective matrix, such as polysaccharides or proteins, they can be shielded from adverse factors during processing and potentially improve their stability in the gastrointestinal tract.

Future research directions should focus on developing specific protection technologies for plant-derived exosomes, such as optimizing microencapsulation methods, exploring novel processing techniques like mild non-thermal processing technologies (e.g., pulsed electric fields), and establishing rapid and accurate exosome quality assessment methods for monitoring stability changes during processing.

### 6.4 Safety considerations

Safety is a critical aspect that requires dedicated attention in future research on PDENs. There may be risks related to immune responses. Since exosomes can carry a variety of biomolecules, there is a possibility that the host immune system might recognize them as foreign invaders and mount an immune response. For example, the presence of certain proteins or nucleic acids in exosomes could potentially trigger an immune reaction, leading to inflammation or other adverse effects. Additionally, the bioactive components in exosomes might interact with the body’s normal physiological processes in unexpected ways, such as interfering with normal cellular signaling pathways.

Currently, there is a lack of clear and comprehensive regulatory frameworks for PDENs in the food and health industries. Different countries may have varying regulations regarding the use of exosomes in food products, which creates challenges for the development and commercialization of PDEN-based products. For example, the classification of PDEN-containing foods (as dietary supplements, novel foods, or regular food products) is still unclear in many regions. This regulatory ambiguity can slow down the progress of research and development, as companies are hesitant to invest in a product with uncertain regulatory status.

Long-term toxicological studies are essential to fully understand the potential health impacts of PDENs. Short-term studies may not be sufficient to detect subtle or cumulative effects over time. For example, repeated exposure to PDENs over an extended period might lead to the accumulation of certain components in the body, which could have unforeseen consequences. These studies should involve different animal models and various doses of PDENs to accurately assess their safety profile.

### 6.5 Development of functional foods

PDENs, with their excellent biocompatibility, stability, and targeting capabilities, are suitable as delivery systems for functional components to enhance their bioavailability. Future applications of PDENs in functional foods may include:

Developing PDEN-fortified beverages. For example, adding blueberry-derived PDENs rich in anthocyanins, which have antioxidant properties, to fruit juices can enhance their antioxidant efficacy (75).

Producing PDEN-encapsulated microcapsules for addition to yogurt, cereals, and other foods. This can improve the stability and absorption rate of functional components. For instance, encapsulating curcumin-rich PDENs in a polysaccharide-based matrix and adding them to yogurt can protect curcumin from degradation and improve its bioavailability. Developing PDEN spray-dried powders that can be directly added to various foods or formulated into solid beverages. Using PDENs to develop novel probiotic products by delivering specific miRNAs to regulate gut microbiota.

During product development, key issues to address include the selection of PDEN sources, optimization of isolation and purification processes, functional evaluation, and compatibility with food matrices. For example, the choice of PDEN source should consider factors such as the type of bioactive components, stability, and availability. The isolation and purification processes need to be optimized to ensure high-quality exosomes with minimal contaminants. Simultaneously, safety assessment and standardization of PDENs are also focal points for future research.

As research progresses, PDENs are expected to become important tools for developing a new generation of functional foods, offering novel approaches to enhance the nutritional and health value of foods.

Therefore, future research should focus on the stability of PDENs during storage, processing, and extraction, while exploring methods to improve their stability and bioactivity. These studies, along with safety considerations, will provide a solid scientific foundation for the application of PDENs in functional foods and human health.

## 7 Conclusion

In summary, after decades of in-depth research and development, PDENs, as an emerging natural bioactive ingredient, have shown enormous potential for application in the fields of food nutrition and health. Its excellent characteristics of low immunogenicity, high stability, and ability to penetrate biological barriers provide new ideas and possibilities for the development of functional foods. These nanoparticles can not only directly promote health as bioactive ingredients, but also serve as carrier systems to achieve precise delivery and release of various nutrients and bioactive compounds.

However, the application of PDENs in the field of functional foods still faces many challenges. In terms of extraction technology, there is currently a lack of simple and reliable methods for large-scale extraction of high-purity PDENs. For example, although traditional ultracentrifugation can obtain PDENs of certain purity, it has problems such as cumbersome operation, long time consumption, and low yield, which are difficult to meet the large-scale production needs of the food industry and limit its wide application in the food industry. In terms of basic research, there is still insufficient research on the surface marker proteins of PDENs, which affects our in-depth understanding of their targeting and biological functions. For example, there are differences in surface proteins of PDENs from different sources, and there is currently a lack of in-depth research on how these differences affect their interactions with cells, distribution, and function *in vivo*. In addition, the mechanism by which PDENs regulate human physiological and pathological processes is still unclear and requires further basic research to clarify. In terms of immune regulation, the specific molecular mechanisms by which PDENs interact with immune cells, activate or inhibit immune responses are still unclear.

Despite these limitations and unresolved issues, the potential value of PDENs in the field of functional foods cannot be underestimated. Its unique biological characteristics and multifunctionality make it a highly promising direction for current and future food science research. Future research should focus on the following actionable directions: optimizing extraction techniques, developing new extraction methods such as affinity chromatography or microfluidic based extraction methods, improving extraction efficiency and purity, and reducing production costs. In the study of food matrix interactions, systematic research is conducted on the interactions between PDENs and different food components (such as proteins, polysaccharides, lipids, etc.) under different processing conditions, to clarify their effects on the stability, biological activity, and function of PDENs, and to provide a basis for the formulation design of functional foods. In terms of bioavailability and safety assessment, human clinical trials and long-term animal experiments will be conducted to comprehensively evaluate the bioavailability and safety of PDENs at different doses and intake methods, and develop safety usage standards. At the same time, it is necessary to strengthen the exploration of the functions of PDENs in immune regulation, metabolic control, anti-inflammatory and antioxidant aspects. For example, by using gene editing technology or proteomic methods, in-depth research can be conducted on the key molecules and signaling pathways through which PDENs exert these functions, providing scientific basis for the development of new functional foods.

Overall, PDENs represent the forefront of innovation in the development of functional foods, and their unique advantages provide new possibilities for improving the nutritional value of food and promoting human health. Although there are still many issues that urgently need to be addressed, through interdisciplinary collaboration and continuous in-depth research, PDENs are expected to become a key factor in promoting the development of the functional food industry and making important contributions to improving the quality of human life.
